# Meta-analysis of risk factors for Parkinson’s disease dementia

**DOI:** 10.1186/s40035-016-0058-0

**Published:** 2016-06-01

**Authors:** Yaqian Xu, Jing Yang, Huifang Shang

**Affiliations:** Department of Neurology, West China Hospital, Sichuan University, 610041 Chengdu, Sichuan China

**Keywords:** Parkinson’s disease, Dementia, Risk factors, Predictors

## Abstract

**Background:**

Parkinson’s disease (PD) is a common heterogeneous neurodegenerative disorder in elder population. Parkinson’s disease dementia (PDD) is one of the most common non-motor manifestations in PD patients. No comprehensive review has been conducted to assess risk factors for PDD.

**Methods:**

A systemic search for studies on PDD risk factors was performed. Cohort and case–control studies that clearly defined PDD and presented relevant data were included. The data were analyzed to generate a pooled effect size and 95 % confidence interval (CI). Publication bias was assessed using the Egger’s test and the Begg’s test.

**Results:**

A systematic search was conducted and yielded 5195 articles. After screening, 25 studies were included in the current analysis. Development of PDD was positively associated with age (odds ratio [OR] 1.07, 95 % CI 1.03-1.13), male (OR 1.33, 95 % CI 1.08-1.64), higher Unified Parkinson’s Disease Rating Scale (UPDRS) part III scores (relative risk [RR] 1.04, 95 % CI 1.01-1.07), hallucination (OR 2.47, 95 % CI 1.36-4.47), REM sleep behavior disorder (RBD) (OR 8.38, 95 % CI 3.87-18.08), smoking (ever vs. never) (RR 1.93, 95 % CI 1.15-3.26) and hypertension (OR 1.57, 95 % CI 1.11-2.22). An inverse association was found between education (RR 0.94, 95 % CI 0.91-0.98) and PDD. Other reported factors, including age of onset, disease duration of PD, Hoehn and Yahr stage and diabetes mellitus were not significantly associated with PDD.

**Conclusions:**

Advanced age, male, higher UPDRS III scores, hallucination, RBD, smoking and hypertension increase the risk of PDD, whereas higher education is a protective factor for PDD.

**Electronic supplementary material:**

The online version of this article (doi:10.1186/s40035-016-0058-0) contains supplementary material, which is available to authorized users.

## Background

Parkinson’s disease (PD), a heterogeneous neurodegenerative disorder in elder population, is characterized by cardinal motor symptoms including bradykinesia, rigidity, tremor and postural instability [1]. Recently, increasing evidence shows that PD is a disease with many non-motor symptoms (NMS) including dementia, sleep disorders, mood disorders, urinary dysfunction, and olfactory disorders [2]. Among NMS, Parkinson disease dementia (PDD) is one of the most common symptoms with a mean prevalence of 31.3 % in PD patients [3]. Among general population, PDD incidence rate is approximately 38.7 to 112.5 per 1000 person-year among several cohort studies conducted in different regions [3, 4]. It has been suggested that PD patients who developed dementia tend to have increased health care burden, declined quality of life and increased mortality [5–7]. However, effective treatment for PDD is currently unclear [8]. Being able to predict PDD development accurately would provide opportunities for intervention as well as novel treatments and might prolong survival [9].

Several demographic, motor and non-motor features have been identified as predictors for PDD. Advanced age is the most common risk factor for dementia and for later diagnosis of PDD in PD patients [10]. More advanced disease stage as well as specific Parkinson subtype, the akinetic-rigid subtype, was found to be associated with increased risk for PDD, whereas the evaluation scales are not coherent [11, 12]. Some studies suggested that REM sleep behavior disorder (RBD), hallucination, mood disorders and olfactory dysfunction are strong predictors for PDD, but the results were not consistent across studies [13–15]. Up to date, no comprehensive meta-analysis on clinical risk factors for PDD has been conducted. A 2014 review on the predictors of PDD by Moore et al. summarized major study results on different risk factors, including clinical predictors, biological predictors, neuroimaging predictors and genetic predictors [9]. In that previous review, the authors presented all possible influences of those factors on PDD, but did not provide quantitative evaluation of the predictors. In order to quantitatively evaluate the effects of different factors on PDD, we conducted this systematic review and meta-analysis via an extensive search of observational studies and a meta-analysis on multiple factors.

## Methods

### Search strategy

We conducted the search according to the Preferred Reporting Items for Systematic Review and Meta-analysis (PRISMA 2009) guideline. We searched MEDLINE and EMBASE database for studies reporting predictors for later diagnosis of PDD. No language restrictions were used. The keywords we selected were: “Parkinson Disease” AND “Dementia” AND “Risk” OR “Predict” OR “Age” OR “Age of Onset” OR “Education” OR “Family history” OR “Hallucination” OR “Sleep Disorders” OR “Constipation” OR “Olfactory Disorders” OR “Color Vision” OR “Depression” OR “Anxiety” OR “Mood Disorders” OR “Erectile Dysfunction” OR “Urinary Dysfunction” OR “Hypertension” OR “Coronary Artery Disease” OR “Head Injury” OR “Diabetes Mellitus” OR “Smoking” OR “Alcohols” OR “Coffee” OR “Pesticides”. We also hand searched the reference lists of relevant reviews and articles with required data for missed references. The final search was carried out on December 1, 2015.

### Inclusion criteria

We included articles that met the predefined criteria: 1) cohort or case–control studies assessed at least one risk factor preceding a later diagnosis of PDD; 2) compared PDD patients with PD patients who did not develop dementia; 3) clearly stated diagnostic criteria for PD and PDD, and carried out by an experienced clinician; 4) reported odds ratio (OR), relative risk (RR) or equivalent values representing risks of developing dementia or case–control studies with cases defined as diagnosed PDD; and 5) reported data that could be easily obtained via questionnaires.

### Exclusion criteria

Reviews, editorials, case reports, commentaries, letters that reported no new data, meta-analysis, handouts, and abstracts were excluded from the study. We excluded studies that: 1) reported on treatment or management of PDD; 2) reported a diseases other than PDD or PD; 3) studied only young onset PD; 4) did not use a PD non-demented group as comparable group to PDD group or did not provide adequate data on the comparable group; 5) were twin studies; 6) reported one predictor repeatedly in one study population (if >1 paper reported on one study population, we chose the larger one, and where population is equal, we chose the most recent one); 7) reported on predictors that were not easily available in most clinical settings (i.e. questionnaires designed for a certain population or country); 8) reported only uncommon genetic risk factors; 9) reported measures other than OR, RR or equivalent values, or from which an OR could not be calculated. Two authors (X.Y. and J.Y.) independently evaluated the eligibility of all studies, and if there was disagreement between authors, the articles were further evaluated by a third author (H.S.) and discussed in detail until an agreement has been reached.

### Data extraction and quality assessment

Study characteristics, PDD diagnostic criteria, a risk estimate of the main study finding, and secondary findings were extracted using a unified form. We did not include studies that reported dementia before or within one year to the onset of PD, since these cases did not fulfill the diagnostic criteria for PDD and were more likely cases of dementia with Lewy bodies (DLB). If studies did not report OR, RR or equivalent measures, raw data were screened to determine whether ORs could be calculated. When the studies reported both the crude OR/RRs and the adjusted OR/RRs, the adjusted figures were extracted. We calculated a quality score to assess the quality of the studies according to the Newcastle-Ottawa Scale (NOS). Length of time that any predictor precedes the diagnosis of PDD was not analyzed in the study due to inconsistent reporting.

### Statistical analysis

We combined the reported risks first separately for case–control and cohort studies, and second for all studies together, in cases where two or more studies reported on the same factor. The data were analyzed to generate a pooled effect size and 95 % confidence interval (CI). We examined the heterogeneity across studies using the I^2^ statistic [16, 17]. Where statistically significant heterogeneity was found (*p* < 0.05), we used randomized effects model to combine results. We assessed publication bias using the Egger’s test and the Begg’s test, and constructed funnel plot in order to visualize any possible asymmetry [18]. Two-tailed *P* values less than 0.05 were considered statistically significant. All analyses were performed using Stata version 12.0 (StataCorp, College Station, TX).

## Results

**Fig. 1:**
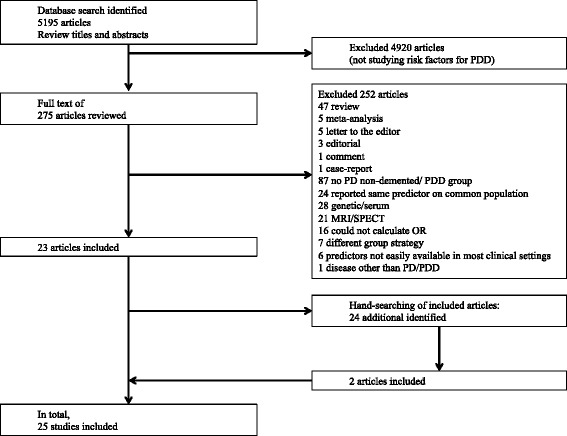
Flowchart of study selection; PDD = Parkinson Disease Dementia

The electronic search yielded 5195 articles, all of which were reviewed by titles and abstracts. Full text of 278 articles were reviewed, of which 23 articles met the inclusion criteria. We also hand searched the references of the articles, 2 of which were included into the analysis. Finally, a total of 25 articles were included in the meta-analysis. Full details of the studies included were provided (Additional files 1 and 2). The selection process is shown in a flow diagram (Fig. 1).

**Fig. 2:**
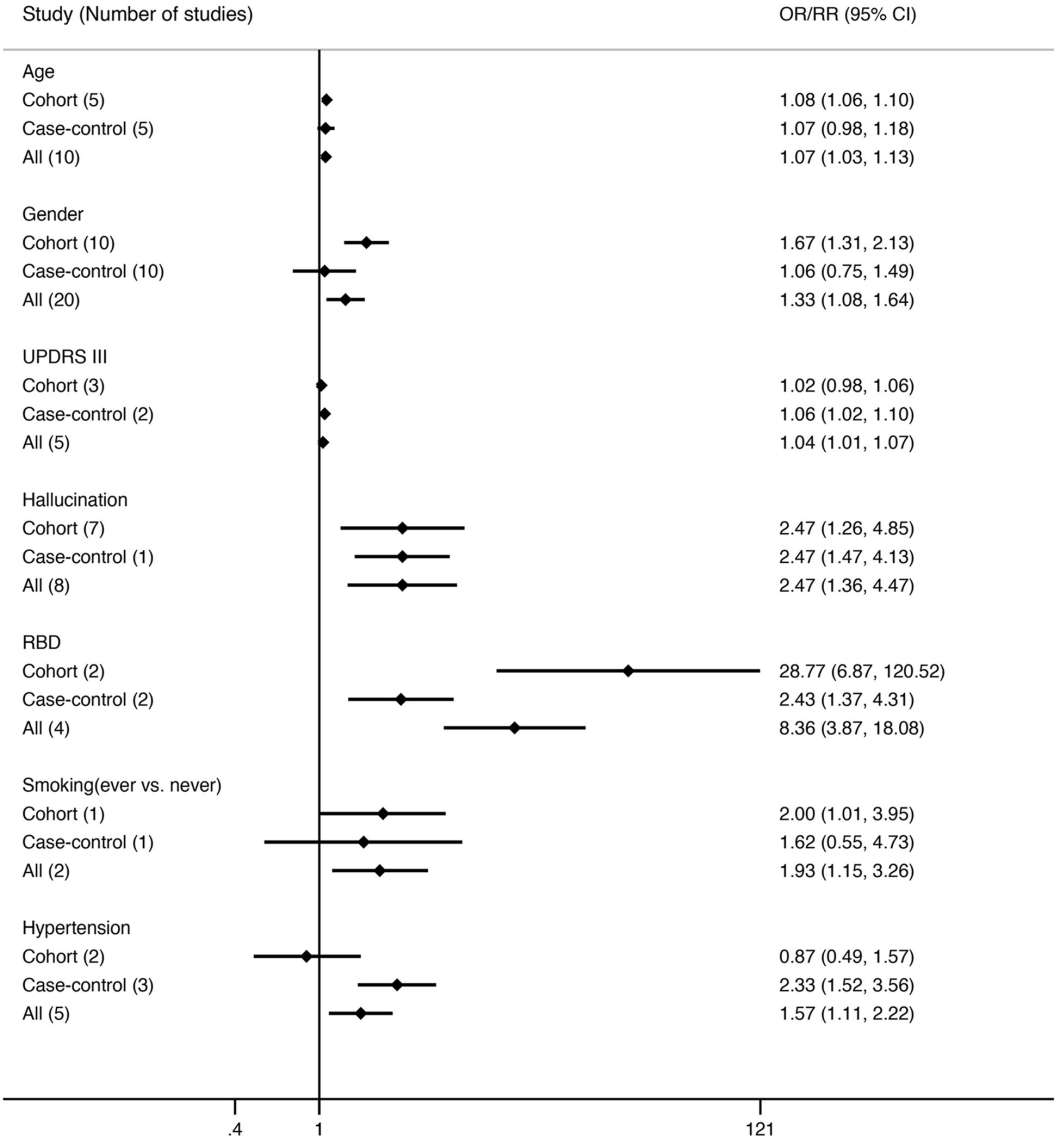
Factors that show significant positive association with PDD; RBD = REM sleep behavior disorder; CI = confidence interval; RR = relative risk; OR = odds ratio

**Fig. 3:**
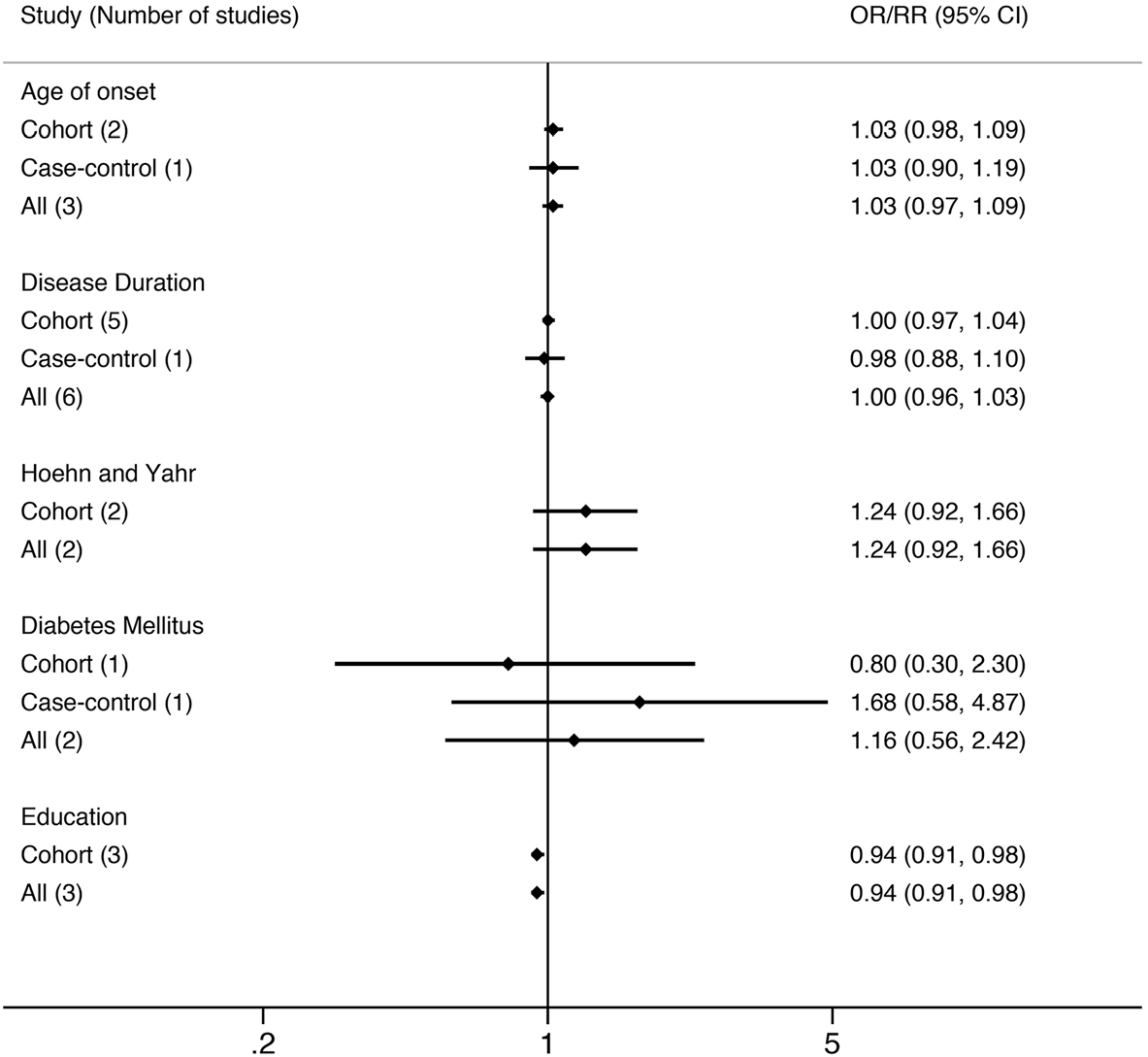
Factor that shows significant negative association with PDD and factors that show no significant association with PDD; CI = confidence interval; RR = relative risk; OR = odds ratio

We found age (OR 1.07, 95 % CI 1.03-1.13), gender (Male) (OR 1.33, 95 % CI 1.08-1.64) and hypertension (OR 1.57, 95 % CI 1.11-2.22), to be significantly associated with later diagnosis of PDD (Fig. 2), whereas age of onset (AOO) (RR 1.03, 95 % CI 0.97-1.09), disease duration of PD (RR 1.00, 95 % CI 0.96-1.03), and type 2 diabetes mellitus (OR 1.16, 95 % CI 0.58-2.42) were not associated with risk of PDD (Fig. 3). Although education was only reported in three cohort studies, it was the only factor we found to decrease PDD risk (RR 0.94, 95 % CI 0.91-0.98) (Fig. 3). Lifestyle related risk factors were poorly reported. Previous and current smokers were reported to have increased risk of developing PDD (RR 1.88, 95 % CI 1.06-3.34) comparing with non-smoking PD population. However, alcohol consumption (RR 1.1, 95 % CI 0.6-2.2) and coffee consumption (RR 0.9, 95 % CI 0.5-1.8), reported in one study population, was not a significant risk factor for PDD [19].

Two general scales evaluating PD patient’s motor features were included in this study. Our result showed that higher score in Unified Parkinson’s Disease Rating Scale (UPDRS) part III (RR 1.04, 95 % CI 1.01-1.07) increased the risk of PDD, but higher Hoehn and Yahr stage (RR 1.24, 95 % CI 0.92-1.66) was not associated with the development PDD. Studies analyzing the association between PD motor subtypes and PDD were not included in the present meta-analysis because of limited data. Two common non-motor symptoms of PD patients, hallucination (OR 2.47, 95 % CI 1.36-4.47) and RBD (OR 8.38, 95 % CI 3.87-18.08), were both strong predictors of PDD. Single studies also reported positive association between PDD and family history of dementia (first-degree relatives), urinary dysfunction, impaired color vision or orthostatic blood pressure drop and no significant association between family history of PD (first-degree relatives), exposure to pesticides, occupational exposure to chemicals and PDD [13, 15, 20]. Details of factors not included in the meta-analysis were provided (Additional file 3).

### Assessment of publication bias

The funnel plot for each included factors were individually examined visually. The shape was presented essentially symmetrical in age, AOO, gender, smoking, disease duration, education, UPDRS III, hallucination, RBD, hypertension, diabetes mellitus and Hoehn and Yahr stage, which was proved by Begg’s and Egger’s test.

## Discussion

This study identified 12 individual predictors that have potential value in screening for PDD. The identified risk factors include demographic characteristics, lifestyle factors, non-motor features of PD, and widely accepted scales evaluating PD. Some of the factors may present pathogenic importance while others could represent the relationship between symptoms and cognitive decline. Together, these factors tend to be markers preceding diagnosis of PDD in PD patients. Of the identified factors, 7 factors were significant predictors for subsequent diagnosis of PDD, the understanding of which may contribute to higher quality of care and improve quality of life in PD patients.

Age, AOO and disease duration were all common risk factors for PDD. However, since these three factors are interdependent, their individual effect on PDD is under debate. One study comparing early and late onset PD patients suggested that late onset PD group presents with more severe impaired sensory abilities, sleep disorders and dementia [21]. Another study adjusted cofounding found that among the three factors, only age remained an independent risk factor for PDD [22]. Similarly, we found that older age had a significant influence on later diagnosis of PDD, while AOO and disease duration was not associated with PDD. Our study suggested that advanced age as a risk factor for PDD may be independent from PD related time factors, like AOO or disease duration. Many neuropathological studies have provided evidences on the effect of aging. Recent studies with α-synuclein immunostaining found a strong association between the age-related increase of Lewy bodies in cortical areas and the development of PDD [23, 24]. Another study found that cortical amyloid-β deposition and aging together might be associated with PDD [25]. Few other studies revealed that MAPT genotype, related with tau transcription, has a strong influence on the risk of PDD [26, 27]. Goris et al. found that, among MAPT haplotypes, PD patients with H1 homozygotes had an increased rate of cognitive decline, which was dependent on age [28, 29]. These age-related pathological processes together may increase the risk of PDD with aging.

We also found male to be a risk factor for PDD. However, some studies suggested that there might be no relationship between gender and PDD if potential confounding factors, including age, history of dementia, smoking and number of siblings, were adjusted [20, 22]. In the present meta-analysis, all data included for the factor gender were unadjusted data. Therefore, our study illustrated that male, before adjustment, is a positive predictor for PDD, but provided no evidence on the results after confounding factors were adjusted. The only protective factor we found in the current meta-analysis is higher education. Similar with our results, one study found that when compared with elementary school level, PDD patients with university level education were at lower risk of developing dementia [30]. One possible explanation is that education might modify the risk of cognitive decline by greater functional brain reserve in PD patients [31]. However, in a recent systematic review looking into education and dementia in Alzheimer’s disease (AD), the authors suggested that although lower education is associated with greater risk of dementia, the findings varied by region, age, gender and ethnicity [32]. The association between education and dementia in PD also need further studying with confounding adjusted.

A recent meta-analysis on risk factors for PD suggested that smoking, alcohol and coffee consumption decreases the risk of PD [33]. In the current meta-analysis we found that a history of smoking increased the risk of dementia in PD by almost two fold. On the other hand, alcohol and coffee consumption has been reported to have no significant association with PDD [19]. Smoking, different from alcohol and coffee consumption, stand as an independent risk factor for PDD. One possible mechanism is related with vascular factors. We found that hypertension was associated with PDD while diabetes was not significantly associated with PDD. Because smoking is also a risk factor for hypertension and that hypertension is related with AD-type pathologies in dementia, it is possible that smoking affect dementia in PD via vascular route [34, 35]. However, after adjustment for the possibility of confounding vascular factors in one included study, smoking still exists as an increased risk for PDD [19]. Biologically, greater depletion of cholinergic cells in the nucleus basalis of Meynert has been observed in PDD [36], yet the up-regulation of central nicotine acetylcholine receptors by nicotine contrasts the mechanism [37, 38]. Another published meta-analysis found that smoking was also a cause of cognitive decline in AD patients as well as in patients with other dementia, in which the authors suggested that non-smokers have lower inflammation or oxidative stress that may lead to a reduction in cognitive decline [39, 40].

We found that higher UPDRS III score was positively related with later diagnosis of PDD, whereas Hoehn and Yahr stage was not significantly associated with PDD. This result suggests that severe motor dysfunction is associated with PDD risk, and that the risk is more likely associated with individual motor dysfunctions. In a study that discovered no significant association between UPDRS III and PDD, researchers found that within the UPDRS III section, gait dysfunction was strongly associated with eventual development of dementia [13]. Gait dysfunction is important for the classification of the postural instability and gait difficulties (PIGD) subtype, and several longitudinal studies have discovered that 25 % to 64.9 % of PIGD PD patients would be diagnosed with PDD by the end of follow-up [11, 12]. Also, researchers have found that increased loss of cholinergic nuclei may relate with both cognitive decline and motor features including rigidity, gait, and balance [12, 41]. However, only two studies were evaluated regarding Hoehn and Yahr stage, the result should be interpreted with caution.

We discovered RBD and hallucination are strongly associated with later diagnosis of PDD. Several studies suggested that patients with RBD had a higher rate of MCI at baseline and a shorter duration towards diagnosis of dementia [42–44]. Cholinergic deficit due to degeneration of ascending pathway that took place in both RBD and dementia with Lewy bodies might be the cause [45]. Visual hallucinations were also discovered to have positive association with cognitive impairment in early PD [46]. In terms of mechanism, pathological studies in PD indicated that visual hallucination might share common limbic pathology with cognitive decline and dementia [14]. In functional MRI assessment, preceding image recognition, patients with visual hallucinations have reduced activation in ventral/lateral visual associated cortices [47]. However, a reverse relation was found in previous study suggesting that cognitive impairment at baseline precedes later development of hallucination [48]. Both RBD and hallucination were also associated with MCI, an important predictor for PDD [49, 50], in early Parkinson disease [42, 46]. None of the studies included in our meta-analysis adjusted RBD or hallucination for baseline cognitive impairment, therefore the causal relation is unclear from the present study.

### Limitations

We only selected factors that were easily obtained in primary care environment. Variables that used less common inventories, like color vision and olfactory dysfunction, were not included in the analysis. Also, genetic tests have been excluded from the analysis for similar reasons. Mild cognitive impairment, an important risk factor of incidence dementia in PD [50, 51], was not included in the meta-analysis because most studies on mild cognitive impairment used a different group strategy by separating the participants into PD-normal cognition, PD-mild cognitive impairment and PDD group, which differs from most of other PDD risk factor studies. Motor subtypes were not analyzed in this meta-analysis also because of differences in group strategy.

Not all studies adjusted risk factors for confounders, and those adjusted were mostly adjusted for different confounders. We included both factors unadjusted and factors adjusted where possible, which may have increased the degree of significance for some risk factors. Few factors were reported in studies to be positively associated or not associated with later diagnosis of PDD but without specified OR or RR to be extracted. Though we have calculated OR or RR where possible, there may still be data neglected.

Statistically significant heterogeneity was found in 8 of the meta-analyses performed in our study. Two risk factors, age and AOO, were found to have high heterogeneity (I^2^ > 75 %). Gender, education, hallucination, UPDRS III, RBD and hypertension were found to have moderate heterogeneity (50 % < I^2^ < 75 %). The presence of heterogeneity was as expected because of the differences in the characteristics of studies, the length of follow-up, study population scale, population characteristics, diagnostic criteria used and whether factors were crude or adjusted. The follow-up period in the cohort studies and the diagnostic criteria for PDD in both cohort and case–control studies varied, we did not adjust these factors in this meta-analysis due to limited number of included studies. Thus, the results of this analysis should be interpreted cautiously, especially for those factors that were reported in less than three studies and that were with high heterogeneities.

In the present meta-analysis, we included studies with NOS score higher than 5, which ensured study quality. Also, we performed sub-group analysis by different study design in order to minimize heterogeneity. We combined analytical results according to the heterogeneity analysis, and did not find significant publication bias. Therefore, our results were considered to be robust.

## Conclusions

This is the first systematic review and meta-analysis on widely evaluated risk factors of PDD. This study found advanced age, male, high UPDRS III scores, presence of hallucination, presence of RBD, ever smoking and history of hypertension are positive predictors to later diagnosis of PDD, whereas education is a protective factor of PDD. This study laid the foundation to future comparative assessment on risk factors for PDD, and lead to a better understanding of PDD risks.

## Abbreviations

AD, Alzheimer’s disease; AOO, Age of onset; CI, Confidence interval; DLB, Dementia with lewy bodies; NOS, Newcastle-Ottawa scale; OR, Odds ratio; PD, Parkinson’s disease; PDD, Parkinson’s disease dementia; PIGD, Postural instability and gait difficulties; PRISMA, Preferred reporting items for systematic review and meta-analysis; RBD, REM sleep behavior disorder; RR, Relative risk; UPDRS, Unified Parkinson’s Disease Rating Scale.

## Additional files

Additional file 1: Details of studies included in the meta-analysis. This file included details of all studies included in this meta-analysis, categorized by risk factors. Details including first author, year of publication, country, study design, number of participants and NOS scores. (DOCX 119 kb) Additional file 2: Details of the study results. This file provided detailed results of meta-analysis of each risk factor. (DOCX 159 kb) Additional file 3: Details of factors not included in the meta-analysis. This file provided data of risk factors for Parkinson’s disease dementia that were mentioned in literature, but were not included in the meta-analysis due to limited number of studies or differences in study design. (DOCX 93 kb)
