# Supplementary material for: Meta-analysis of risk factors for Parkinson’s disease dementia
Source: Transl Neurodegener. 2016 Jun 1;5:11. doi: 10.1186/s40035-016-0058-0 (PMC4890279; doi:10.1186/s40035-016-0058-0)
Supplement: Additional file 1: — Details of studies included in the meta-analysis. This file included details of all studies included in this meta-analysis, categorized by risk factors. Details including first author, year of publication, country, study design, number of participants and NOS scores. (DOCX 119 kb) [file 40035_2016_58_MOESM1_ESM.docx]

**Additional file 1 - Details of studies included in the meta-analysis**

PDD definitions

DSM III-R = Diagnostic and Statistical Manual of Mental Disorders, revised third edition^[[1]](#footnote-1)^

DSM IV = the Diagnostic and Statistical Manual of Mental Disorders, Forth Edition^[[2]](#footnote-2)^

DSM IV-TR = Diagnostic and Statistical Manual of Mental Disorders, Fourth Edition, Text Revision^[[3]](#footnote-3)^

MDS = Clinical Diagnostic Criteria for Dementia associated with Parkinson’s Disease^[[4]](#footnote-4)^

MMSE = Mini-Mental State Examination

SCOPA-COG = SCales for Outcomes in PArkinson's disease-COGnition^[[5]](#footnote-5)^

1.Age

| **Ref** | **First Author** | **Year** | **Country** | **Study Design** | **Follow-up Duration** | **PDD** | **M** | **F** | **Mean Age** | **PDND** | **M** | **F** | **Mean Age** | **PDD Definition** | **NOS** |
| --- | --- | --- | --- | --- | --- | --- | --- | --- | --- | --- | --- | --- | --- | --- | --- |
| 1 | Zhu | 2014 | Netherland | Cohort | 4.83(0.81) | 129 | 79 | 50 | 66.46 | 277 | 180 | 97 | 58.19 | SCOPA-COG≤22 | 7 |
| 2 | Aarsland | 2007 | USA and Norway | Cohort | 2.9(3.0) | 123 | - | - | - | 364 | - | - | - | DSM III-R | 7 |
| 3 | Anang | 2014 | Canada | Cohort | 4.4 | 27 | 22 | 5 | 70.48 | 53 | 29 | 24 | 63.6 | MDS | 7 |
| 4 | Nomura | 2013 | Japan | Cohort | 1.78(0.90) | 12 | - | - | - | 70 | - | - | - | MDS | 7 |
| 5 | Fitts* | 2015 | US | Cohort | 3.13 | 26 | - | - | - | 106 | - | - | - | MDS | 9 |
| 15 | Schelp | 2012 | Brazil | Case-control | 0 | 19 | - | - | - | 60 | - | - | - | Neuropsychological schedule for dementia diagnosis | 6 |
| 16 | Slawek | 2013 | Poland | Case-control | 0 | 57 | 29 | 28 | 67.98 | 135 | 62 | 73 | 61.91 | MDS | 7 |
| 17 | Pondal | 1996 | Spain | Case-control | 0 | 15 | 3 | 12 | 72.5 | 55 | 31 | 24 | - | DSM III-R | 6 |
| 24 | Zoccolella | 2009 | Italy | Case-control | 0 | 42 | 27 | 15 | 71.2 | 79 | 45 | 34 | 65.4 | DSM IV-TR | 8 |

* Adjusted for gender, education, duration of PD, hallucination, apathy and UPDRS-III score

2.Age of onset

| **Ref** | **First Author** | **Year** | **Country** | **Study Design** | **Follow-up Duration** | **PDD** | **M** | **F** | **Mean Age** | **PDND** | **M** | **F** | **Mean Age** | **PDD Definition** | **NOS** |
| --- | --- | --- | --- | --- | --- | --- | --- | --- | --- | --- | --- | --- | --- | --- | --- |
| 1 | Zhu | 2014 | Netherland | Cohort | 4.83(0.81) | 129 | 79 | 50 | 66.46 | 277 | 180 | 97 | 58.19 | SCOPA-COG≤22 | 7 |
| 2 | Aarsland | 2007 | USA and Norway | Cohort | 2.9(3.0) | 123 | - | - | - | 364 | - | - | - | DSM III-R | 7 |
| 18 | Camicioli | 2005 | Canada | Case-control | 0 | 28 | 22 | 6 | - | 19 | 11 | 8 | - | DSM IV | 6 |

3. Disease duration

| **Ref** | **First Author** | **Year** | **Country** | **Study Design** | **Follow-up Duration** | **PDD** | **M** | **F** | **Mean Age** | **PDND** | **M** | **F** | **Mean Age** | **PDD Definition** | **NOS** |
| --- | --- | --- | --- | --- | --- | --- | --- | --- | --- | --- | --- | --- | --- | --- | --- |
| 1 | Zhu | 2014 | Netherland | Cohort | 4.83(0.81) | 129 | 79 | 50 | 66.46 | 277 | 180 | 97 | 58.19 | SCOPA-COG≤22 | 7 |
| 14 | Levy | 2002 | USA | Cohort | 3.6(2.2) | 52 | 29 | 23 | 74.6 | 128 | 54 | 74 | 69.5 | DSM III-R | 9 |
| 3 | Anang | 2014 | Canada | Cohort | 4.4 | 27 | 22 | 5 | 70.48 | 53 | 29 | 24 | 63.6 | MDS | 7 |
| 4 | Nomura | 2013 | Japan | Cohort | 1.78(0.90) | 12 | - | - | - | 70 | - | - | - | MDS | 7 |
| 5 | Fitts* | 2015 | US | Cohort | 3.13 | 26 | - | - | - | 106 | - | - | - | MDS | 9 |
| 15 | Schelp | 2012 | Brazil | Case-control | 0 | 19 | - | - | - | 60 | - | - | - | Neuropsychological schedule for dementia diagnosis | 6 |

* Adjusted for age, gender, education, hallucination, apathy and UPDRS-III score

4. Gender

| **Ref** | **First Author** | **Year** | **Country** | **Study Design** | **Follow-up Duration** | **PDD** | **M** | **F** | **Mean Age** | **PDND** | **M** | **F** | **Mean Age** | **PDD Definition** | **NOS** |
| --- | --- | --- | --- | --- | --- | --- | --- | --- | --- | --- | --- | --- | --- | --- | --- |
| 12 | Mahieux | 1998 | France | Cohort | 3.5 | 19 | 14 | 5 | 72.2 | 62 | 32 | 30 | 65.4 | DSM III-R | 7 |
| 3 | Anang | 2014 | Canada | Cohort | 4.4 | 27 | 22 | 5 | 70.48 | 53 | 29 | 24 | 63.6 | MDS | 7 |
| 1 | Zhu | 2014 | Netherland | Cohort | 4.83(0.81) | 129 | 79 | 50 | 66.46 | 277 | 180 | 97 | 58.19 | SCOPA-COG≤22 | 7 |
| 9 | Baba | 2012 | Japan | Cohort | 3 | 10 | 8 | 2 | 67 | 34 | 14 | 20 | 64.7 | MDS | 7 |
| 2 | Aarsland | 2007 | USA and Norway | Cohort | 2.9(3.0) | 123 | - | - | - | 364 | - | - | - | DSM III-R | 7 |
| 7 | Compta | 2013 | Spain | Cohort | 1.5 | 11 | 7 | 4 | 74 | 16 | 12 | 4 | 67 | MDS | 7 |
| 5 | Fitts | 2015 | US | Cohort | 3.13 | 26 | - | - | - | 106 | - | - | - | MDS | 9 |
| 6 | Sanyal | 2014 | India | Cohort | 7 | 121 | 97 | 24 | - | 92 | 78 | 14 | - | DSM III R | 7 |
| 10 | Gago | 2009 | Portigal | Cohort | 6 | 7 | 5 | 2 | 67.29 | 17 | 10 | 7 | 62.24 | DSM IV | 7 |
| 11 | Olde | 2014 | Netherland | Cohort | 7.03(1.17) | 19 | 15 | 4 | 66 | 44 | 24 | 20 | 60.9 | MDS | 6 |
| 15 | Schelp | 2012 | Brazil | Case-control | 0 | 19 | - | - | - | 60 | - | - | - | Neuropsychological schedule for dementia diagnosis | 6 |
| 17 | Pondal | 1996 | Spain | Case-control | 0 | 15 | 3 | 12 | 72.5 | 55 | 31 | 24 | - | DSM III-R | 6 |
| 18 | Camicioli | 2005 | Canada | Case-control | 0 | 28 | 22 | 6 | - | 19 | 11 | 8 | - | DSM IV | 6 |
| 24 | Zoccolella | 2009 | Italy | Case-control | 0 | 42 | 27 | 15 | 71.2 | 79 | 45 | 34 | 65.4 | DSM IV-TR | 8 |
| 19 | Llebaria | 2008 | Spain | Case-control | 0 | 35 | 18 | 17 | 77.6 | 57 | 36 | 21 | 67.3 | DSM IV-TR | 6 |
| 25 | Riedel | 2010 | Germany | Case-control | 0 | 397 | - | - | 73.6 | 1052 | - | - | 69.5 | DSM IV | 7 |
| 20 | Archibald | 2011 | UK | Case-control | 0 | 26 | 22 | 4 | 71.2 | 64 | 42 | 22 | 70.2 | MDS | 6 |
| 16 | Slawek | 2013 | Poland | Case-control | 0 | 57 | 29 | 28 | 67.98 | 135 | 62 | 73 | 61.91 | MDS | 7 |
| 22 | Rosengarten | 2010 | Germany | Case-control | 0 | 116 | 76 | 40 | - | 59 | 39 | 20 | - | DSM IV-TR | 6 |
| 23 | Burn | 2006 | UK | Case-control | 0 | 42 | 27 | 15 | 73.1 | 40 | 30 | 10 | 75.4 | DSM IV | 6 |

5. Education

| **Ref** | **First Author** | **Year** | **Country** | **Study Design** | **Follow-up Duration** | **PDD** | **M** | **F** | **Mean Age** | **PDND** | **M** | **F** | **Mean Age** | **PDD Definition** | **NOS** |
| --- | --- | --- | --- | --- | --- | --- | --- | --- | --- | --- | --- | --- | --- | --- | --- |
| 1 | Zhu | 2014 | Netherland | Cohort | 4.83(0.81) | 129 | 79 | 50 | 66.46 | 277 | 180 | 97 | 58.19 | SCOPA-COG≤22 | 7 |
| 2 | Aarsland | 2007 | USA and Norway | Cohort | 2.9(3.0) | 123 | - | - | - | 364 | - | - | - | DSM III-R | 7 |
| 5 | Fitts* | 2015 | US | Cohort | 3.13 | 26 | - | - | - | 106 | - | - | - | MDS | 9 |

* Adjusted for age, gender, duration of PD, hallucination, apathy and UPDRS-III score

6. Hoehn and Yahr stage

| **Ref** | **First Author** | **Year** | **Country** | **Study Design** | **Follow-up Duration** | **PDD** | **M** | **F** | **Mean Age** | **PDND** | **M** | **F** | **Mean Age** | **PDD Definition** | **NOS** |
| --- | --- | --- | --- | --- | --- | --- | --- | --- | --- | --- | --- | --- | --- | --- | --- |
| 1 | Zhu | 2014 | Netherland | Cohort | 4.83(0.81) | 129 | 79 | 50 | 66.46 | 277 | 180 | 97 | 58.19 | SCOPA-COG≤22 | 7 |
| 3 | Anang | 2014 | Canada | Cohort | 4.4 | 27 | 22 | 5 | 70.48 | 53 | 29 | 24 | 63.6 | MDS | 7 |

7. UPDRS III

| **Ref** | **First Author** | **Year** | **Country** | **Study Design** | **Follow-up Duration** | **PDD** | **M** | **F** | **Mean Age** | **PDND** | **M** | **F** | **Mean Age** | **PDD Definition** | **NOS** |
| --- | --- | --- | --- | --- | --- | --- | --- | --- | --- | --- | --- | --- | --- | --- | --- |
| 3 | Anang | 2014 | Canada | Cohort | 4.4 | 27 | 22 | 5 | 70.48 | 53 | 29 | 24 | 63.6 | MDS | 7 |
| 2 | Aarsland | 2007 | USA and Norway | Cohort | 2.9(3.0) | 123 | - | - | - | 364 | - | - | - | DSM III-R | 7 |
| 5 | Fitts* | 2015 | US | Cohort | 3.13 | 26 | - | - | - | 106 | - | - | - | MDS | 9 |
| 19 | Llebaria | 2008 | Spain | Case-control | 0 | 35 | 18 | 17 | 77.6 | 57 | 36 | 21 | 67.3 | DSM IV-TR | 6 |
| 24 | Zoccolella# | 2009 | Italy | Case-control | 0 | 42 | 27 | 15 | 71.2 | 79 | 45 | 34 | 65.4 | DSM IV-TR | 8 |

* Adjusted for age, gender, duration of PD, hallucination, apathy and education

# Adjusted for age at entry, sex, UPDRS III score, education, hypertension and B12 and folate levels

8. REM sleep behavior disorder

| **Ref** | **First Author** | **Year** | **Country** | **Study Design** | **Follow-up Duration** | **PDD** | **M** | **F** | **Mean Age** | **PDND** | **M** | **F** | **Mean Age** | **PDD Definition** | **NOS** |
| --- | --- | --- | --- | --- | --- | --- | --- | --- | --- | --- | --- | --- | --- | --- | --- |
| 3 | Anang | 2014 | Canada | Cohort | 4.4 | 27 | 22 | 5 | 70.48 | 53 | 29 | 24 | 63.6 | MDS | 7 |
| 4 | Nomura | 2013 | Japan | Cohort | 1.78(0.90) | 12 | - | - | - | 70 | - | - | - | MDS | 7 |
| 20 | Archibald | 2011 | UK | Case-control | 0 | 26 | 22 | 4 | 71.2 | 64 | 42 | 22 | 70.2 | MDS | 6 |
| 21 | Marion | 2008 | UK | Case-control | 0 | 13 | - | - | - | 52 | - | - | - | DSM IV | 6 |

9. Hallucination

| **Ref** | **First Author** | **Year** | **Country** | **Study Design** | **Follow-up Duration** | **PDD** | **M** | **F** | **Mean Age** | **PDND** | **M** | **F** | **Mean Age** | **PDD Definition** | **NOS** |
| --- | --- | --- | --- | --- | --- | --- | --- | --- | --- | --- | --- | --- | --- | --- | --- |
| 1 | Zhu | 2014 | Netherland | Cohort | 4.83(0.81) | 129 | 79 | 50 | 66.46 | 277 | 180 | 97 | 58.19 | SCOPA-COG≤22 | 7 |
| 3 | Anang | 2014 | Canada | Cohort | 4.4 | 27 | 22 | 5 | 70.48 | 53 | 29 | 24 | 63.6 | MDS | 7 |
| 13 | Aarsland | 2003 | Norway | Cohort | 8 | 122 | 55 | 67 | - | 102 | 52 | 49 | - | DSM III R | 7 |
| 4 | Nomura | 2013 | Japan | Cohort | 1.78(0.90) | 12 | - | - | - | 70 | - | - | - | MDS | 7 |
| 7 | Compta | 2013 | Spain | Cohort | 1.5 | 11 | 7 | 4 | 74 | 16 | 12 | 4 | 67 | MDS | 7 |
| 5 | Fitts* | 2015 | US | Cohort | 3.13 | 26 | - | - | - | 106 | - | - | - | MDS | 9 |
| 6 | Sanyal | 2014 | India | Cohort | 7 | 121 | 97 | 24 | - | 92 | 78 | 14 | - | DSM III R | 7 |
| 25 | Riedel# | 2010 | Germany | Case-control | 0 | 397 | - | - | 73.6 | 1052 | - | - | 69.5 | DSM IV | 7 |

* Adjusted for age, gender, education, duration of PD, apathy and UPDRS-III score

# Adjusted for age

10. Hypertension

| **Ref** | **First Author** | **Year** | **Country** | **Study Design** | **Follow-up Duration** | **PDD** | **M** | **F** | **Mean Age** | **PDND** | **M** | **F** | **Mean Age** | **PDD Definition** | **NOS** |
| --- | --- | --- | --- | --- | --- | --- | --- | --- | --- | --- | --- | --- | --- | --- | --- |
| 8 | Levy* | 2002 | USA | Cohort | 3.6(2.2) | 52 | 29 | 23 | 74.6 | 128 | 54 | 74 | 69.5 | DSM III-R | 9 |
| 7 | Compta | 2013 | Spain | Cohort | 1.5 | 11 | 7 | 4 | 74 | 16 | 12 | 4 | 67 | MDS | 7 |
| 25 | Riedel^@^ | 2010 | Germany | Case-control | 0 | 397 | - | - | 73.6 | 1052 | - | - | 69.5 | DSM IV | 7 |
| 24 | Zoccolella# | 2009 | Italy | Case-control | 0 | 42 | 27 | 15 | 71.2 | 79 | 45 | 34 | 65.4 | DSM IV-TR | 8 |
| 15 | Schelp | 2012 | Brazil | Case-control | 0 | 19 | - | - | - | 60 | - | - | - | Neuropsychological schedule for dementia diagnosis | 6 |

* Adjusted for age at baseline, gender, years of education, duration of PD, and total UPDRS motor score at baseline

# Adjusted for age at entry, sex, UPDRS III score, education, hypertension and B12 and folate levels

^@^ Adjusted for age

11. Diabetes mellitus

| **Ref** | **First Author** | **Year** | **Country** | **Study Design** | **Follow-up Duration** | **PDD** | **M** | **F** | **Mean Age** | **PDND** | **M** | **F** | **Mean Age** | **PDD Definition** | **NOS** |
| --- | --- | --- | --- | --- | --- | --- | --- | --- | --- | --- | --- | --- | --- | --- | --- |
| 8 | Levy* | 2002 | USA | Cohort | 3.6(2.2) | 52 | 29 | 23 | 74.6 | 128 | 54 | 74 | 69.5 | DSM III-R | 9 |
| 15 | Schelp | 2012 | Brazil | Case-control | 0 | 19 | - | - | - | 60 | - | - | - | Neuropsychological schedule for dementia diagnosis | 6 |

* Adjusted for age at baseline, gender, years of education, duration of PD, and total UPDRS motor score at baseline

12. Smoking (ever vs. never)

| **Ref** | **First Author** | **Year** | **Country** | **Study Design** | **Follow-up Duration** | **PDD** | **M** | **F** | **Mean Age** | **PDND** | **M** | **F** | **Mean Age** | **PDD Definition** | **NOS** |
| --- | --- | --- | --- | --- | --- | --- | --- | --- | --- | --- | --- | --- | --- | --- | --- |
| 8 | Levy* | 2002 | USA | Cohort | 3.6(2.2) | 52 | 29 | 23 | 74.6 | 128 | 54 | 74 | 69.5 | DSM III-R | 9 |
| 22 | Rosengarten | 2010 | Germany | Case-control | 0 | 116 | 76 | 40 | - | 59 | 39 | 20 | - | DSM IV-TR | 6 |

* Adjusted for age at baseline, gender, years of education, duration of PD, and total UPDRS motor score at baseline

**List of references**

1. Zhu K, van Hilten JJ, Marinus J. Predictors of dementia in Parkinson's disease; findings from a 5-year prospective study using the SCOPA-COG. Parkinsonism & related disorders. 2014;20:980-5.

2. Aarsland D, Kvaloy JT, Andersen K, Larsen JP, Tang MX, Lolk A, et al. The effect of age of onset of PD on risk of dementia. Journal of neurology. 2007;254:38-45.

3. Anang JB, Gagnon JF, Bertrand JA, Romenets SR, Latreille V, Panisset M, et al. Predictors of dementia in Parkinson disease: A prospective cohort study. Neurology. 2014;83:1253-60.

4. Nomura T, Inoue Y, Kagimura T, Nakashima K. Clinical significance of REM sleep behavior disorder in Parkinson's disease. Sleep medicine. 2013;14:131-5.

5. Fitts W, Weintraub D, Massimo L, Chahine L, Chen-Plotkin A, Duda JE, et al. Caregiver report of apathy predicts dementia in Parkinson's disease. Parkinsonism & related disorders. 2015;21:992-5.

6. Sanyal J, Banerjee TK, Rao VR. Dementia and cognitive impairment in patients with Parkinson's disease from India: a 7-year prospective study. American journal of Alzheimer's disease and other dementias. 2014;29:630-6.

7. Compta Y, Pereira JB, Rios J, Ibarretxe-Bilbao N, Junque C, Bargallo N, et al. Combined dementia-risk biomarkers in Parkinson's disease: a prospective longitudinal study. Parkinsonism & related disorders. 2013;19:717-24.

8. Levy G, Tang MX, Cote LJ, Louis ED, Alfaro B, Mejia H, et al. Do risk factors for Alzheimer's disease predict dementia in Parkinson's disease? An exploratory study. Movement disorders. 2002;17:250-7.

9. Baba T, Kikuchi A, Hirayama K, Nishio Y, Hosokai Y, Kanno S, et al. Severe olfactory dysfunction is a prodromal symptom of dementia associated with Parkinson's disease: a 3 year longitudinal study. Brain. 2012;135:161-9.

10. Gago MF, Garrett MC, Fonseca MR, Rosas MJ, Simoes MF, Vieira S, et al. How do cognitive and axial motor signs correlate in Parkinson's disease? A 6-year prospective study. Journal of neurology. 2009;256:1655-62.

11. Olde Dubbelink KT, Hillebrand A, Twisk JW, Deijen JB, Stoffers D, Schmand BA, et al. Predicting dementia in Parkinson disease by combining neurophysiologic and cognitive markers. Neurology. 2014;82:263-70.

12. Mahieux F, Fenelon G, Flahault A, Manifacier MJ, Michelet D, Boller F. Neuropsychological prediction of dementia in Parkinson's disease. Journal of neurology, neurosurgery, and psychiatry. 1998;64:178-83.

13. Aarsland D, Andersen K, Larsen JP, Lolk A, Kragh-Sorensen P. Prevalence and characteristics of dementia in Parkinson disease: an 8-year prospective study. Archives of neurology. 2003;60:387-92.

14. Levy G, Jacobs DM, Tang MX, Cote LJ, Louis ED, Alfaro B, et al. Memory and executive function impairment predict dementia in Parkinson's disease. Movement disorders. 2002;17:1221-6.

15. Schelp AO, Mendes-Chiloff CL, Bazan R, Paduan VC, Pioltini AB. Metabolic syndrome and dementia associated with Parkinson's disease: impact of age and hypertension. Arquivos de neuro-psiquiatria. 2012;70:114-8.

16. Slawek J, Roszmann A, Robowski P, Dubaniewicz M, Sitek EJ, Honczarenko K, et al. The impact of MRI white matter hyperintensities on dementia in Parkinson's disease in relation to the homocysteine level and other vascular risk factors. Neuro-degenerative diseases. 2013;12:1-12.

17. Pondal M, Del Ser T, Bermejo F. Anticholinergic therapy and dementia in patients with Parkinson's disease. Journal of neurology. 1996;243:543-6.

18. Camicioli R, Rajput A, Rajput M, Reece C, Payami H, Hao C, et al. Apolipoprotein E epsilon4 and catechol-O-methyltransferase alleles in autopsy-proven Parkinson's disease: relationship to dementia and hallucinations. Movement disorders. 2005;20:989-94.

19. Llebaria G, Pagonabarraga J, Kulisevsky J, Garcia-Sanchez C, Pascual-Sedano B, Gironell A, et al. Cut-off score of the Mattis Dementia Rating Scale for screening dementia in Parkinson's disease. Movement disorders. 2008;23:1546-50.

20. Archibald NK, Clarke MP, Mosimann UP, Burn DJ. Visual symptoms in Parkinson's disease and Parkinson's disease dementia. Movement disorders. 2011;26:2387-95.

21. Marion MH, Qurashi M, Marshall G, Foster O. Is REM sleep behaviour disorder (RBD) a risk factor of dementia in idiopathic Parkinson's disease? Journal of neurology. 2008;255:192-6.

22. Rosengarten B, Dannhardt V, Burr O, Pohler M, Rosengarten S, Oechsner M, et al. Neurovascular coupling in Parkinson's disease patients: effects of dementia and acetylcholinesterase inhibitor treatment. Journal of Alzheimer's disease : JAD. 2010;22:415-21.

23. Burn DJ, Rowan EN, Allan LM, Molloy S, O'Brien JT, McKeith IG. Motor subtype and cognitive decline in Parkinson's disease, Parkinson's disease with dementia, and dementia with Lewy bodies. Journal of neurology, neurosurgery, and psychiatry. 2006;77:585-9.

24. Zoccolella S, dell'Aquila C, Abruzzese G, Antonini A, Bonuccelli U, Canesi M, et al. Hyperhomocysteinemia in levodopa-treated patients with Parkinson's disease dementia. Movement disorders. 2009;24:1028-33.

25. Riedel O, Klotsche J, Spottke A, Deuschl G, Forstl H, Henn F, et al. Frequency of dementia, depression, and other neuropsychiatric symptoms in 1,449 outpatients with Parkinson's disease. Journal of neurology. 2010;257:1073-82.

1. Diagnostic and statistical manual of mental disorders. Rev 3rd ed. Washington, DC: American Psychiatric Press; 1987. [↑](#footnote-ref-1)
2. Diagnostic and statistical manual of mental disorders. 4th ed. Washington, DC: American Psychiatric Association, 1994. [↑](#footnote-ref-2)
3. Diagnostic and statistical manual of mental disorders. 4th ed, text revision. Washington, DC: American Psychiatric Association, 2000. [↑](#footnote-ref-3)
4. Emre M, et al. Clinical diagnostic criteria for dementia associated with Parkinson's disease. Movement disorders. 2007;22 (12):1689-1707. [↑](#footnote-ref-4)
5. Verbaan D, Marinus J, Visser M, van Rooden SM, Stiggelbout AM, Middelkoop HA, van Hilten JJ. Cognitive impairment in Parkinson's disease. Journal of neurology, neurosurgery, and psychiatry. 2007;78 (11):1182-1187. [↑](#footnote-ref-5)
