# Supplementary material for: Meta-analysis of risk factors for Parkinson’s disease dementia
Source: Transl Neurodegener. 2016 Jun 1;5:11. doi: 10.1186/s40035-016-0058-0 (PMC4890279; doi:10.1186/s40035-016-0058-0)
Supplement: Additional file 2: — Details of the study results. This file provided detailed results of meta-analysis of each risk factor. (DOCX 159 kb) [file 40035_2016_58_MOESM2_ESM.docx]

**Additional file 2 - Details of the study results**

CI= Confidence Interval; OR= Odd Ratio; RR= Relative Risk

1. Age

1.1 Cohort studies (Fixed model)

1.2 Case-control studies (Random model)

2. Age of onset

2.1 Cohort studies (Random model)

2.2 Case-control studies

| **First Author** | **Year** | **Study design** | **OR/RR** | **CI lower limit** | **CI upper limit** |
| --- | --- | --- | --- | --- | --- |
| Camicioli | 2005 | Case-control | 1.03 | 0.90 | 1.19 |

3. Disease duration

3.1 Cohort studies (Fixed model)

3.2 Case-control studies

| **First Author** | **Year** | **Study design** | **OR/RR** | **CI lower limit** | **CI upper limit** |
| --- | --- | --- | --- | --- | --- |
| Schelp | 2012 | Case-control | 0.98 | 0.88 | 1.10 |

4. Gender

4.1 Cohort studies (Fixed model)

4.2 Case-control studies (Random model)

5. Education

5.1 Cohort studies (Fixed model)

6. Hoehn and Yahr stage

6.1 Cohort studies (Fixed model)

7. Hallucination

7.1 Cohort studies (Random model)

7.2 Case-control studies

| **First Author** | **Year** | **Study design** | **OR/RR** | **CI lower limit** | **CI upper limit** |
| --- | --- | --- | --- | --- | --- |
| Riedel | 2010 | Case-control | 2.47 | 1.47 | 4.13 |

8. UPDRS III

8.1 Cohort studies (Random model)

8.2 Case-control studies (Fixed model)

9. REM sleep behavior disorder

9.1 Cohort studies (Fixed model)

9.2 Case-control studies (Fixed model)

10. Hypertension

10.1 Cohort studies (Fixed model)

10.2 Case-control studies (Fixed model)

11. Type II diabetes mellitus

11.1 Cohort studies

| **First Author** | **Year** | **Study design** | **OR/RR** | **CI lower limit** | **CI upper limit** |
| --- | --- | --- | --- | --- | --- |
| Levy | 2002 | Cohort | 0.80 | 0.30 | 2.30 |

11.2 Case-control studies

| **First Author** | **Year** | **Study design** | **OR/RR** | **CI lower limit** | **CI upper limit** |
| --- | --- | --- | --- | --- | --- |
| Schelp | 2012 | Case-control | 1.68 | 0.58 | 4.87 |

12. Smoking (ever vs. never)

12.1 Cohort studies

| **First Author** | **Year** | **Study design** | **OR/RR** | **CI lower limit** | **CI upper limit** |
| --- | --- | --- | --- | --- | --- |
| Levy | 2002 | Cohort | 2.00 | 1.00 | 3.90 |

12.2 Case-control studies

| **First Author** | **Year** | **Study design** | **OR/RR** | **CI lower limit** | **CI upper limit** |
| --- | --- | --- | --- | --- | --- |
| Rosengarten | 2010 | Case-control | 1.87 | 0.84 | 4.12 |
