# Supplementary material for: Meta-analysis of risk factors for Parkinson’s disease dementia
Source: Transl Neurodegener. 2016 Jun 1;5:11. doi: 10.1186/s40035-016-0058-0 (PMC4890279; doi:10.1186/s40035-016-0058-0)
Supplement: Additional file 3: — Details of factors not included in the meta-analysis. This file provided data of risk factors for Parkinson’s disease dementia that were mentioned in literature, but were not included in the meta-analysis due to limited number of studies or differences in study design. (DOCX 93 kb) [file 40035_2016_58_MOESM3_ESM.docx]

**Additional file 3 - Details of factors not included in the meta-analysis**

1. Family history of dementia (first-degree relatives)

| **Ref** | **First Author** | **Year** | **Study design** | **OR/RR** | **CI lower limit** | **CI upper limit** | **Evaluation scales** |
| --- | --- | --- | --- | --- | --- | --- | --- |
| 1 | Marder | 1990 | Case-control | 6.47 | 1.22 | 22.04 | Yes:no |

2. Family history of PD (first-degree relatives)

| **Ref** | **First Author** | **Year** | **Study design** | **OR/RR** | **CI lower limit** | **CI upper limit** | **Evaluation scales** |
| --- | --- | --- | --- | --- | --- | --- | --- |
| 1 | Marder | 1990 | Case-control | 0.67 | 0.118 | 1.903 | Yes:no |

3. Alcohol consumption

| **Ref** | **First Author** | **Year** | **Study design** | **OR/RR** | **CI lower limit** | **CI upper limit** | **Evaluation scales** |
| --- | --- | --- | --- | --- | --- | --- | --- |
| 2 | Levy | 2002 | Cohort | 1.1 | 0.6 | 2.2 | Ever vs. nonusers |

4. Coffee consumption

| **Ref** | **First Author** | **Year** | **Study design** | **OR/RR** | **CI lower limit** | **CI upper limit** | **Evaluation scales** |
| --- | --- | --- | --- | --- | --- | --- | --- |
| 2 | Levy | 2002 | Cohort | 0.9 | 0.5 | 1.8 | Ever vs. nonusers |

5. Exposure to pesticides

| **Ref** | **First Author** | **Year** | **Study design** | **OR/RR** | **CI lower limit** | **CI upper limit** | **Evaluation scales** |
| --- | --- | --- | --- | --- | --- | --- | --- |
| 1 | Marder | 1990 | Case-control | 0.65 | 0.18 | 1.708 | Yes:no |

6. Occupational exposure to chemicals

| **Ref** | **First Author** | **Year** | **Study design** | **OR/RR** | **CI lower limit** | **CI upper limit** | **Evaluation scales** |
| --- | --- | --- | --- | --- | --- | --- | --- |
| 1 | Marder | 1990 | Case-control | 0.657 | 0 | 2.079 | Yes:no |

7. Color vision impairment

| **Ref** | **First Author** | **Year** | **Study design** | **OR/RR** | **CI lower limit** | **CI upper limit** | **Evaluation scales** |
| --- | --- | --- | --- | --- | --- | --- | --- |
| 3 | Anang | 2014 | Cohort | 1.01 | 1 | 1.02 | F-M 100 error score* |

* Farnsworth-Munsell 100 Test

8. Urinary dysfunction

| **Ref** | **First Author** | **Year** | **Study design** | **OR/RR** | **CI lower limit** | **CI upper limit** | **Evaluation scales** |
| --- | --- | --- | --- | --- | --- | --- | --- |
| 3 | Anang | 2014 | Cohort | 1.84 | 1.01 | 3.34 | Yes:no |

9. Depression

| **Ref** | **First Author** | **Year** | **Study design** | **OR/RR** | **CI lower limit** | **CI upper limit** | **Evaluation scales** |
| --- | --- | --- | --- | --- | --- | --- | --- |
| 3 | Anang | 2014 | Cohort | 2.27 | 1.15 | 4.46 | UPDRS 1.3* |
| 4 | Zhu | 2014 | Cohort | 1.034 | 0.987 | 1.084 | Beck depression inventory |
| 5 | Jacobs | 1995 | Cohort | 1.11 | 1.02 | 1.21 | Hamilton depression rating scale |

* Unified Parkinson's Disease Rating Scale part 1.3

10. Olfactory dysfunction

| **Ref** | **First Author** | **Year** | **Study design** | **OR/RR** | **CI lower limit** | **CI upper limit** | **Evaluation scales** |
| --- | --- | --- | --- | --- | --- | --- | --- |
| 3 | Anang | 2014 | Cohort | 0.94 | 0.87 | 1.03 | UPSIT-40* |
| 6 | Baba | 2012 | Cohort | 18.7 | 3.1 | 425.2 | OSIT-J score# |

* University of Pennsylvania Smell Identification Test, 40-item version

# Odour stick identification test for Japan

11. Orthostatic blood pressure drop

| **Ref** | **First Author** | **Year** | **Study design** | **OR/RR** | **CI lower limit** | **CI upper limit** | **Evaluation scales** |
| --- | --- | --- | --- | --- | --- | --- | --- |
| 3 | Anang | 2014 | Cohort | 3.16 | 1.60 | 6.51 | Yes:no |

12. Head injury

| **Ref** | **First Author** | **Year** | **Study design** | **OR/RR** | **CI lower limit** | **CI upper limit** | **Evaluation scales** |
| --- | --- | --- | --- | --- | --- | --- | --- |
| 2 | Levy | 2002 | Cohort | 0.9 | 0.4 | 2.2 | Yes:no |

13. Estrogen replacement therapy

| **Ref** | **First Author** | **Year** | **Study design** | **OR/RR** | **CI lower limit** | **CI upper limit** | **Evaluation scales** |
| --- | --- | --- | --- | --- | --- | --- | --- |
| 2 | Levy | 2002 | Cohort | 0.21 | 0.03 | 1.70 | Yes:no |

**References**

1. Marder K, Flood P, Cote L, Mayeux R. A pilot study of risk factors for dementia in Parkinson's disease. Movement disorders. 1990;5:156-61.

2. Levy G, Tang MX, Cote LJ, Louis ED, Alfaro B, Mejia H, et al. Do risk factors for Alzheimer's disease predict dementia in Parkinson's disease? An exploratory study. Movement disorders. 2002;17:250-7.

3. Anang JB, Gagnon JF, Bertrand JA, Romenets SR, Latreille V, Panisset M, et al. Predictors of dementia in Parkinson disease: A prospective cohort study. Neurology. 2014;83:1253-60.

4. Zhu K, van Hilten JJ, Marinus J. Predictors of dementia in Parkinson's disease; findings from a 5-year prospective study using the SCOPA-COG. Parkinsonism & related disorders. 2014;20:980-5.

5. Jacobs DM, Marder K, Cote LJ, Sano M, Stern Y, Mayeux R. Neuropsychological characteristics of preclinical dementia in Parkinson's disease. Neurology. 1995;45:1691-6.

6. Baba T, Kikuchi A, Hirayama K, Nishio Y, Hosokai Y, Kanno S, et al. Severe olfactory dysfunction is a prodromal symptom of dementia associated with Parkinson's disease: a 3 year longitudinal study. Brain. 2012;135:161-9.
